# Weekly, Bi-Weekly and Monthly Journals

**Published:** 1881-01

**Authors:** 


WEEKLY, BI-WEEKLY AND MONTHLY JOURNALS.—We have no hesitation in saying to a correspondent that *we* much prefer a properly conducted *Monthly*, to either a "*Semi-Monthly* or *Weekly* Medical Journal." He might have infered as much from seeing we adopted the *monthly* form in the INDEPENDENT PRACTITIONER. There are to our mind, several good reasons for preferring it. In the first place, it is easier to supply a *quid pro quo* to the subscriber in a monthly, conducted on the principles we have endeavored to pursue with this journal, than in a *weekly* or *bi-weekly* publication.

The science of medicine is making no such rapid progress that a *monthly*, of sixty or seventy pages of well digested facts should not be amply adequate for recording all that is actually new at home and abroad, and likely to prove useful to the profession, during the month! It would be easy enough, of course, to fill to repletion, as many pages every *two* weeks, and even *every* week, as this journal contains, if the discussions of the various medical societies were included with other matter. But it will be remembered, we took ground against publishing such proceedings, unless in exceptional instances, in the early part of this enterprise; and no good reason has occurred for a change of purpose or opinion on that point. On the contrary, an occasional glance at such proceedings as are reported in some of the journals, has only strengthened the opinion we then entertained, that they were *not* calculated, as a rule, to benefit readers in actual practice, We endeavor to procure, and invite for publication, well digested and carefully prepared papers, and brief practical articles, whether read before medical societies or not, and to exclude, as a rule, debates upon such subjects as may be sprung upon, or introduced for discussion, before such bodies. In no society, probably, is the *cacoethes loquendi* so frequently found as in a medical one; and we of course, have no objection to its members thus afflicted, gratifying themselves to their hearts' content; but we do not choose to advertise them, and their verbiage, to the exclusion of other, and *more* useful matter to our readers. We are bound, as in a solemn covenant to our subscribers, to supply them with the *most* valuable and useful information we can obtain for their two dollars a year subscription money, and we have no right to occupy their space in the journal with crude utterances of fourth and fifth rate men, which, as often occurs in the reports of *some* societies, amounts to little else than the merest twaddle. And as we cannot report the statements and arguments of *wiser* and *better* men, without evoking invidiousness in the former class, we prefer not to publish them at all. We are thus enabled to devote more space to the judicious arrangement of our gleanings from other journals, and the introduction of original matter. We have not yet introduced the feature of publishing didactic lectures before classes in the medical schools, as such lectures, even from our most eminent professors, are intended and delivered for the benefit of the college students; and as it is reasonable to suppose that physicians have advanced *somewhat* since their student days, we will confine ourselves to the publication of those lectures only which treat of new discoveries, proving the action of remedies, etc., in future issues of this journal.

